# Digital Unwrapping of the Mummy of King Amenhotep I (1525–1504 BC) Using CT

**DOI:** 10.3389/fmed.2021.778498

**Published:** 2021-12-28

**Authors:** Sahar N. Saleem, Zahi Hawass

**Affiliations:** ^1^Department of Radiology, Kasr Al Ainy Faculty of Medicine, Cairo University, Cairo, Egypt; ^2^Antiquities of Egypt, Cairo, Egypt

**Keywords:** ancient Egypt, mummy, computed tomography, ancient diseases, mummification

## Abstract

The mummy of King Amenhotep I (18th Dynasty c.1525–1504 BC) was reburied by the 21st Dynasty priests at Deir el-Bahari Royal Cache. In 1881 the mummy was found fully wrapped and was one of few royal mummies that have not been unwrapped in modern times. We hypothesized that non-invasive digital unwrapping using CT would provide insights on the physical appearance, health, cause of death, and mummification style of the mummy of King Amenhotep I. We examined the mummy with CT and generated two- and three-dimensional images for the head mask, bandages, and the virtually unwrapped mummy. CT enabled the visualization of the face of Amenhotep I who died around the age of 35 years. The teeth had minimal attrition. There was no CT evidence of pathological changes or cause of death. The body has been eviscerated *via* a vertical left flank incision. The heart is seen in the left hemithorax with an overlying amulet. The brain has not been removed. The mummy has 30 amulets/jewelry pieces including a beaded metallic (likely gold) girdle. The mummy suffered from multiple postmortem injuries likely inflicted by tomb robbers that have been likely treated by 21st Dynasty embalmers. These included fixing the detached head and neck to the body with a resin-treated linen band; covering a defect in the anterior abdominal wall with a band and placing two amulets beneath; placement of the detached left upper limb beside the body and wrapping it to the body. The transversely oriented right forearm is individually wrapped, likely representing the original 18th Dynasty mummification and considered the first known New Kingdom mummy with crossed arms at the chest. The head mask is made of cartonnage and has inlaid stone eyes. The digital unwrapping of the mummy of Amenhotep I using CT sets a unique opportunity to reveal the physical features of the King non-invasively, understand the mummification style early in the 18th Dynasty, and the reburial intervention style by 21st Dynasty embalmers. This study may make us gain confidence in the goodwill of the reburial project of the Royal mummies by the 21st dynasty priests.

## Introduction

Amenhotep I ruled Egypt for about 21 years (c.1525–1504 BC). He was the second king of the 18th Dynasty to ascend the throne after the death of his father Ahmose I. Amenhotep I may have co-reigned with his mother Ahmose-Nefertari (1). The name Amenhotep means: “Amun is satisfied”. His throne name was Djeserkare: “Holy is the Soul of Re”. During his reign, Amenhotep I protected the territories of Egypt; he led a campaign to Kush and an expedition to Libya. Amenhotep I had a peaceful reign that enabled him to focus on the administrative organization and commission building work of temples. The most important temples built by Amenhotep I was the temple of Amun at Karnak, a temple in Nubia at Sai, as well as structures in Upper Egypt at Elephantine, Kom Ombo, Abydos, and the Temple of Nekhbet. After his death, Amenhotep I and his mother were worshiped in Deir El Medina (2, 3).

The original tomb of Amenhotep I has not yet been found in modern times. The mummy of Amenhotep I was discovered in 1881 at Deir el-Bahari Royal Cache in Luxor, where the officials of the 21st Dynasty hid the mummies of several New Kingdom kings and nobles to protect them from tomb robbers. The mummy of Amenhotep I was found wrapped inside a coffin (4). The hieroglyphic inscriptions on the coffin, dockets, confirmed the name of Amenhotep I and recorded the rewrapping of the mummy after being damaged by grave robbers. The mummy of Amenhotep I has been rewrapped twice by the 21st Dynasty's priests: by Pinedjem I, Theban High Priest of Amun, and a decade later by his son Masarharta (5–7).

Shortly after its discovery, the mummy of Amenhotep I was moved from Deir el Bahari to Cairo and was first kept at Boulaq Museum, then moved to a palace in Giza (for Ismail Pasha). In 1902, the Royal mummies, including that of Amenhotep I, were moved to the Egyptian Museum at Tahrir in Cairo. The mummy of Amenhotep I was one of the very few royal mummies that have not been unwrapped by modern Egyptologists. Gaston Maspero, the director of antiquities in Egypt at that time, decided to let the mummy remain untouched because of its perfect wrapping completely covered by garlands and its exquisite face mask. When the coffin of Amenhotep I was opened, a preserved wasp was found, possibly attracted by the smell of garlands, and was trapped (8).

In February of 1932, an X-ray study of the mummy of Amenhotep I was done at the Cairo Egyptian Museum after the removal of the mummy from its coffin. Douglas Derry, professor at the Kasr Al Ainy School of Medicine in Cairo, interpreted the X-ray and estimated the age of death of Amenhotep I to be between 40 and 50 years. Derry recorded residue inside the skull and a small amulet in the middle of the right arm (9). In 1967, the Michigan University expedition X-rayed the mummy of Amenhotep I. The X-rays estimated the age at death of Amenhotep I to be about 25 years. The age estimation was based on the good condition of the teeth with minimal attrition. However, the symphyseal surface which gives a more accurate estimation of age, could not be visualized. The radiological image showed a bead girdle on the King, the right forearm was seen flexed at the elbow and crossed the chest, while the broken left arm rested along the flank. The X-ray examinations of the mummy of King Amenhotep I failed to provide consistent data or detailed information on the mummy (10, 11).

In the plain x-ray examination, the three-dimensional (3D) information of the mummy is projected onto a two-dimensional X-ray film. The result is the superimposition of objects and bones which makes mummy characterization less satisfactory. CT is an advanced form of X-ray that obtains hundreds of thin sections (slices) of the body and provides more detailed reconstructed images of soft tissues as well as bones. CT is a non-invasive modality that has been used to examine the mummies of several ancient Egyptian royals. CT provided greater insight into the condition, mummification, health issues, and cause of death of the mummy (12).

In this study, we hypothesized that the CT study of the wrapped mummy of Amenhotep I would give more insights on the physical appearance, health, cause of death, and mummification of the King.

## Materials and Methods

The mummy of Amenhotep I was located at the time of this study at the Gallery of Royal Mummies in the Cairo Egyptian Museum with the catalog code (JE 26211(b) CG 61058 SR 1/10194).

On May 4, 2019, we transferred the mummy to the multi-detector CT scanning machine (Somatom Emotion 6; Siemens Medical Solutions, Malvern, Pennsylvania, United States) installed on a truck in the garden of the Cairo Egyptian Museum. The mummy was physically inspected. We used the following CT parameters: kVp = 130 effective mAs ranged from 23 to 63; pitch ranged from 0.83 to 1.8; field of view (FOV) from 350 to 500; slice thickness from 0.6 to 1.25 mm; and reconstruction from 0.4 to 0.8 mm. Axial images were created. We used a special visualization software (OsiriX, Pixmeo SARL, Bernex, Switzerland) that automatically created a 3D data set. Once the latter was generated, the digital unwrapping of the mummy began by peeling off virtual layers using scalpel tools and by changing the window levels. We evaluated the CT images for foreign objects and amulets and recorded their location and metric measurement (in mm). We analyzed the CT images of the mummy to assess the preservation status, age at death, and pathologies according to protocols published before (12–16). We measured the CT density of the objects in Hounsfield units (HU) by placing a region of interest (ROI) within the object. The material of the object was determined according to its HU measurements: metal (>2,978 HU); quartz/faience (1,693–2,317 HU), stones (about 2,900–2,500 HU), and fired clay (1,116 HU SD 54.7) (17). We correlated the CT findings of the mummy with the available archaeological data and previous physical and radiological studies.

## Results

### Physical Inspection of the Mummy

**Figure 1:**
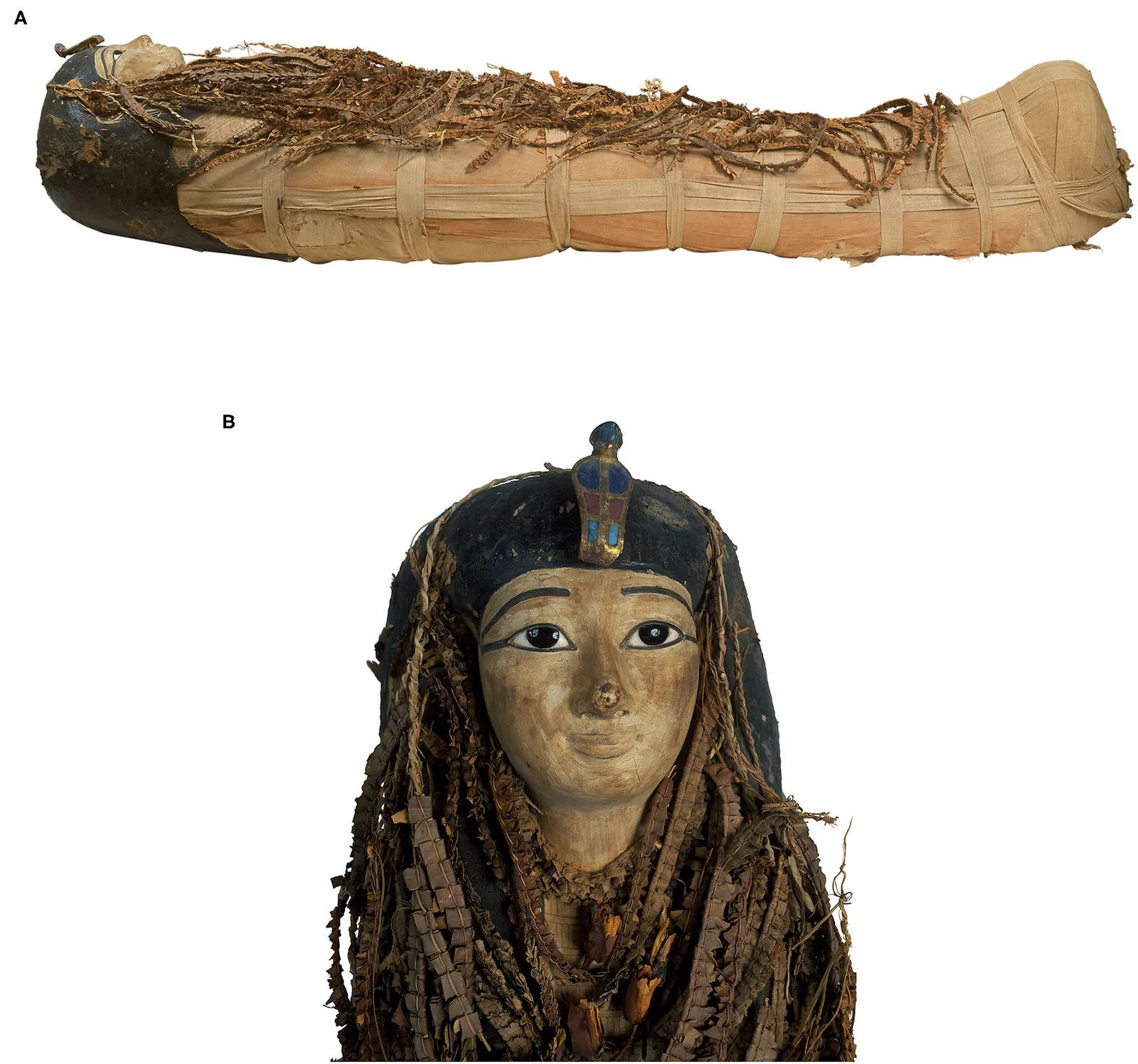
Picture of the mummy of Amenhotep I. **(A)** The picture of the right lateral view of the mummy of Amenhotep I shows the body fully wrapped in linen, covered from head to feet with floral garlands, and wearing a head mask. **(B)** Picture of the head mask of the mummy of Amenhotep I made of painted wood and cartonnage. The face is painted in faint yellow. The contour of the eyes and eyebrows is painted black. The eyes are inlaid with black pupils made of obsidian crystals. On the forehead is a separately carved painted cobra of painted wood, inlaid stones, and cartonnage. The rest of the head mask is partly hidden by floral garlands.

The mummy of Amenhotep I is wrapped in linen and covered from head to feet in floral garlands of red, yellow, and blue color. The head is covered with a mask made out of painted wood and cartonnage. The face is painted pale yellow. The contour of the eyes and eyebrows are painted black. The black eye pupil is made of obsidian crystals. On the forehead is a separately carved painted cobra with inlaid stones. The cartonnage at the chest region is partly hidden by the overlying garlands and could not be inspected (Figure 1).

### CT Study of the Mummy

#### Digital Unwrapping

**Figure 2:**
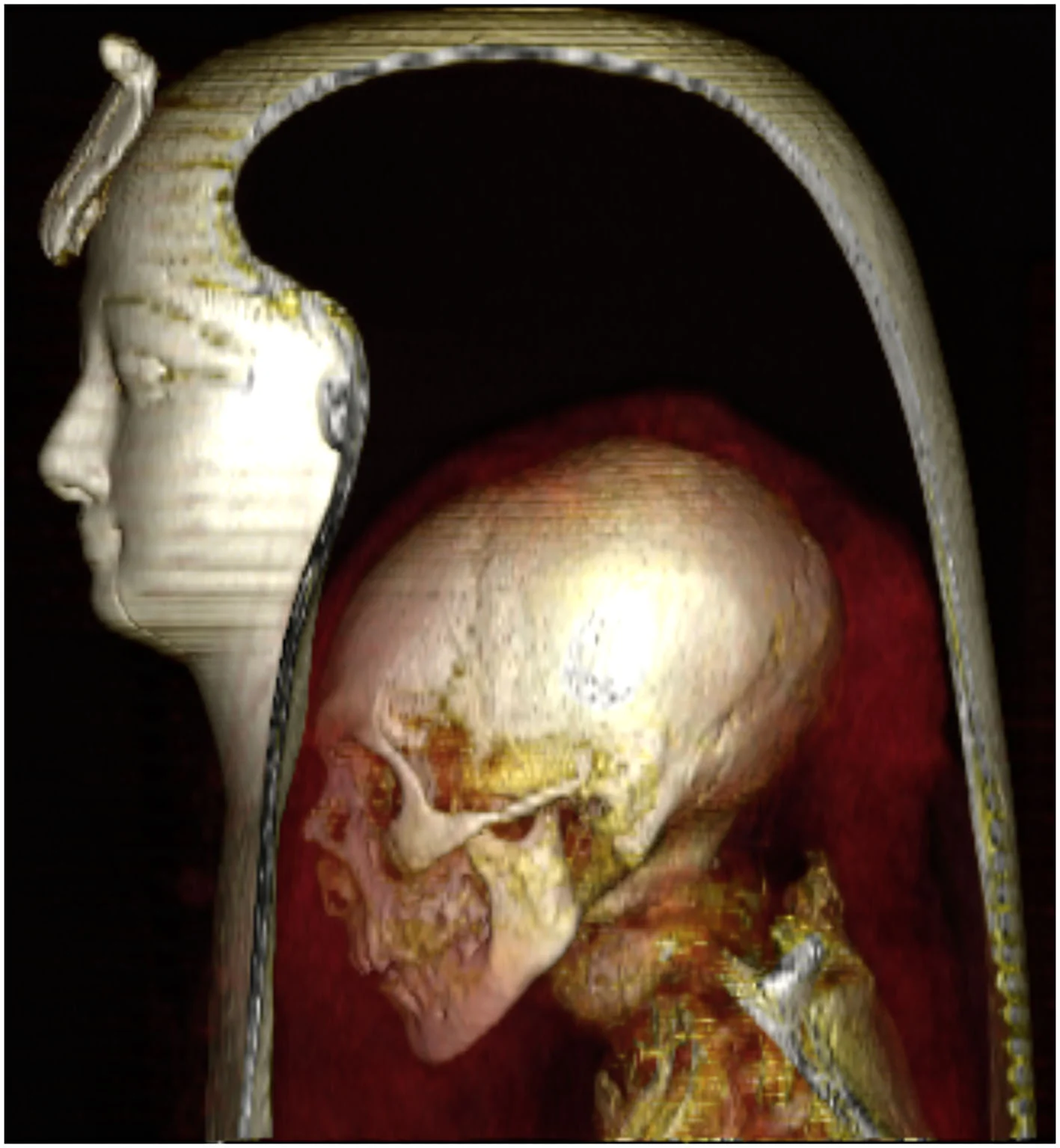
Three-dimensional CT image of the head of the wrapped mummy of Amenhotep I in a left lateral view allows visualization of the component layers: the mask, the head of the mummy, and the surrounding bandages.

The 3D model of the wrapped mummy allowed the visualization of its different component layers: the head mask, the wrapping bandages, and the mummy. The digital unwrapping of the mummy by peeling off virtual layers exposed the exterior and interior of the mummy and allowed us to study it in detail (Figure 2).

**Figure 3:**
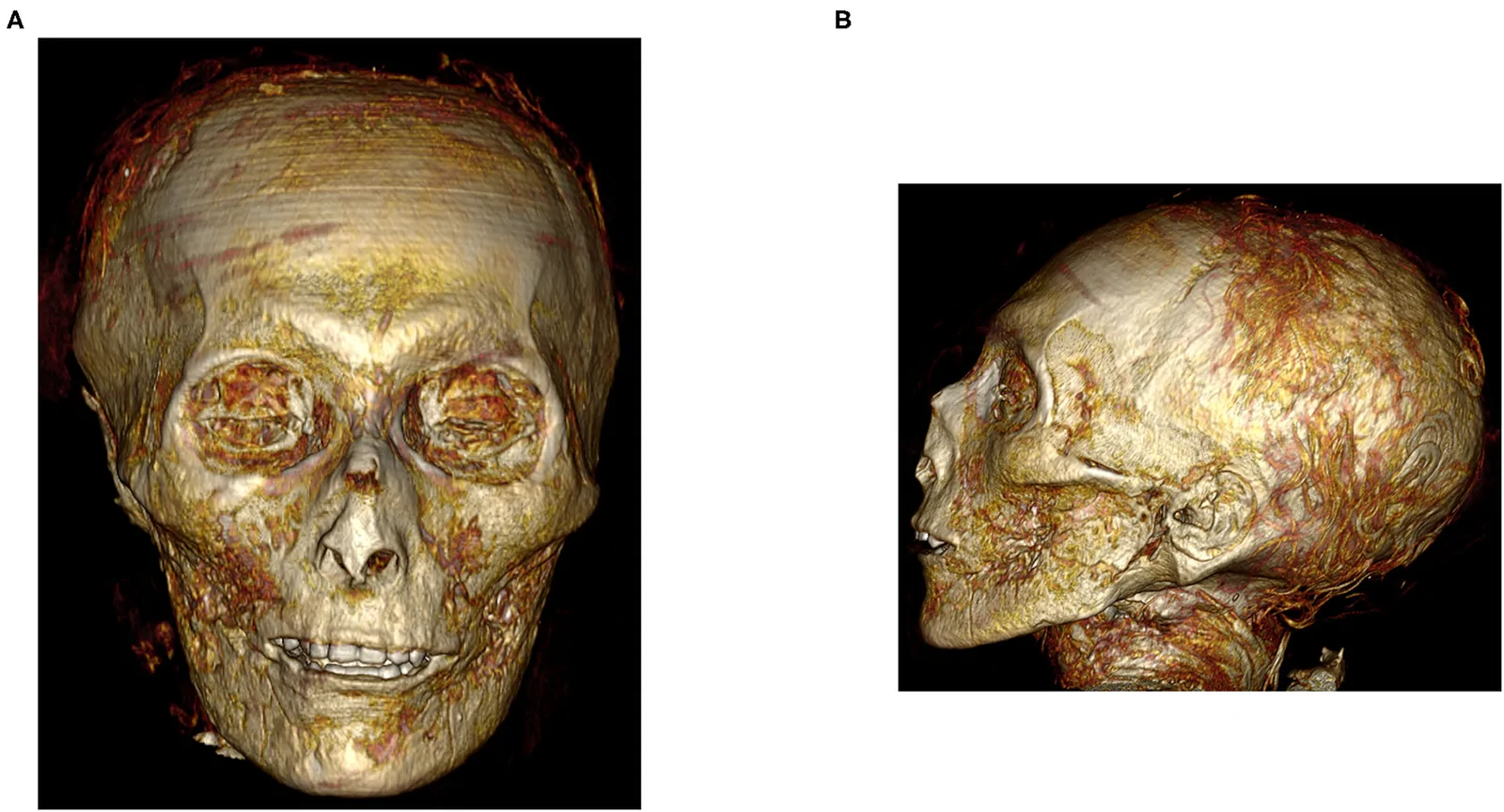
Three-dimensional CT image of the digitally unwrapped face of the mummy Amenhotep I. **(A)** Three-dimensional CT image of the front of the face of Amenhotep I and **(B)** Three-dimensional CT image of the left profile of the face of Amenhotep I show an oval face with a narrow chin, small narrow nose flattened by the bandages, mildly protruding upper teeth, sunken eyes, collapsed cheeks, pierced lobule of the left ear, and few coiled hair locks.

The mummy of Amenhotep I has an oval face with sunken eyes and collapsed cheeks. The nose is small, narrow, and flattened. The upper teeth are mildly protruding. The chin is narrow. The ears are small; a small piercing is noted in the lobule of the left ear. Few coiled hair locks are seen at the back and sides of the head (Figure 3).

#### Preservation Status

**Figure 4:**
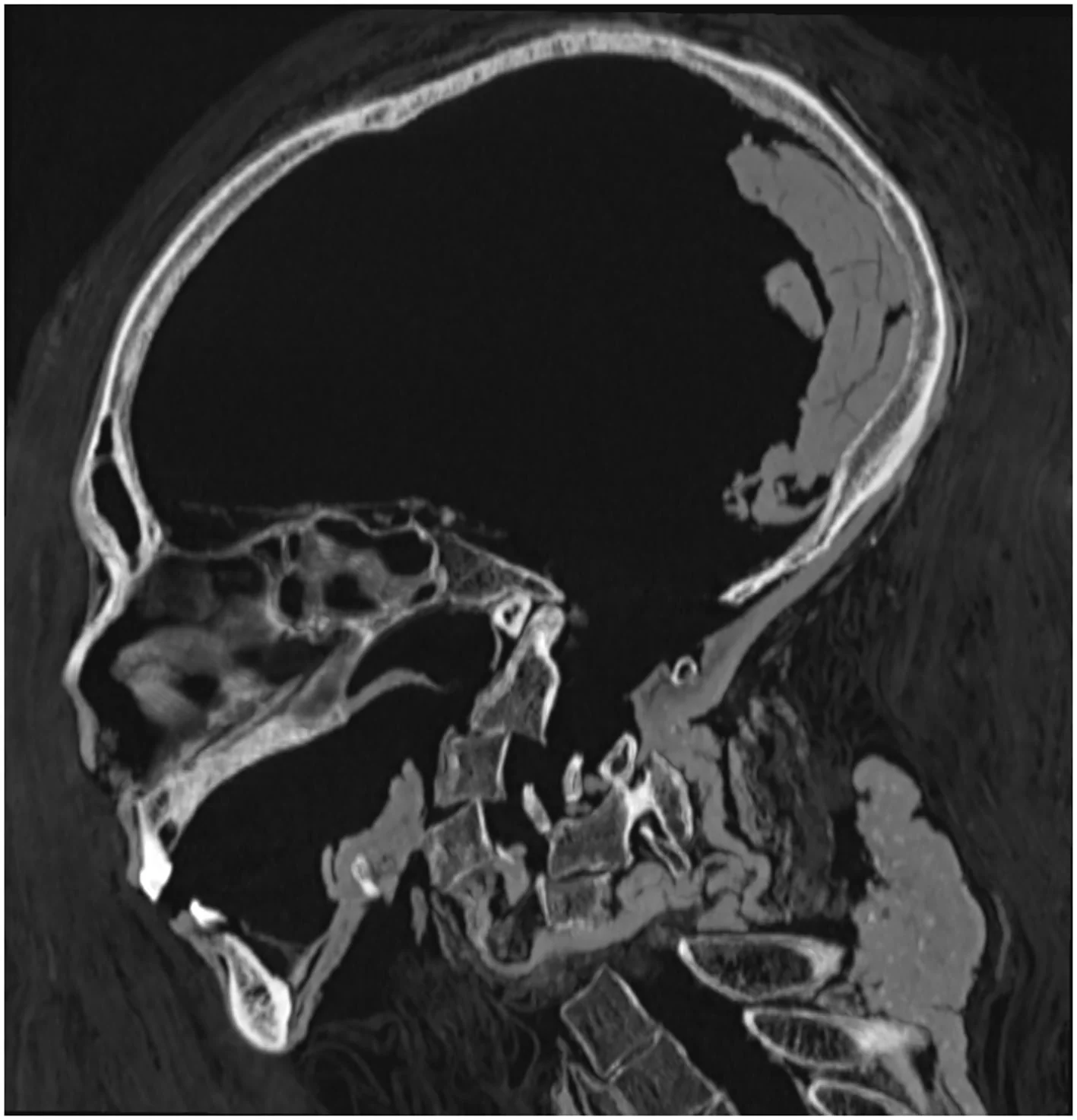
Mid-sagittal CT image of the head and neck of the mummy of Amenhotep I shows an intact cribriform plate and the preserved desiccated brain rests at the back of the skull. Fractured cervical spine with malrotation of the lower three cervical vertebrae. A linen band of linen treated with resin wrapped the fractured cervical spine and fixed the detached head with the dorsal spine.

The mummy of Amenhotep I is in a general good preservation condition. Multiple postmortem injuries are identified including:

Neck fractures and decapitation: A complete transverse fracture of the cervical spine at C4-5 caused decapitation. A complete fracture at the C7-T1 level is noted with dislocation and rotation of the three lower cervical vertebrae (C5, C6, and C7). Resin is noted in the break between the seventh cervical vertebra (C7) and the first thoracic vertebra (T1) (Figure 4).

The right hand is dislocated at the wrist; no bones are missing. The right hand is displaced anterior to the transversely oriented forearm. The left upper limb is dislocated from the shoulder and elbow and lies beside the body with the hand broken off. Only three flexed fingers are available in the left hand and the carpal bones are missing.

**Figure 5:**
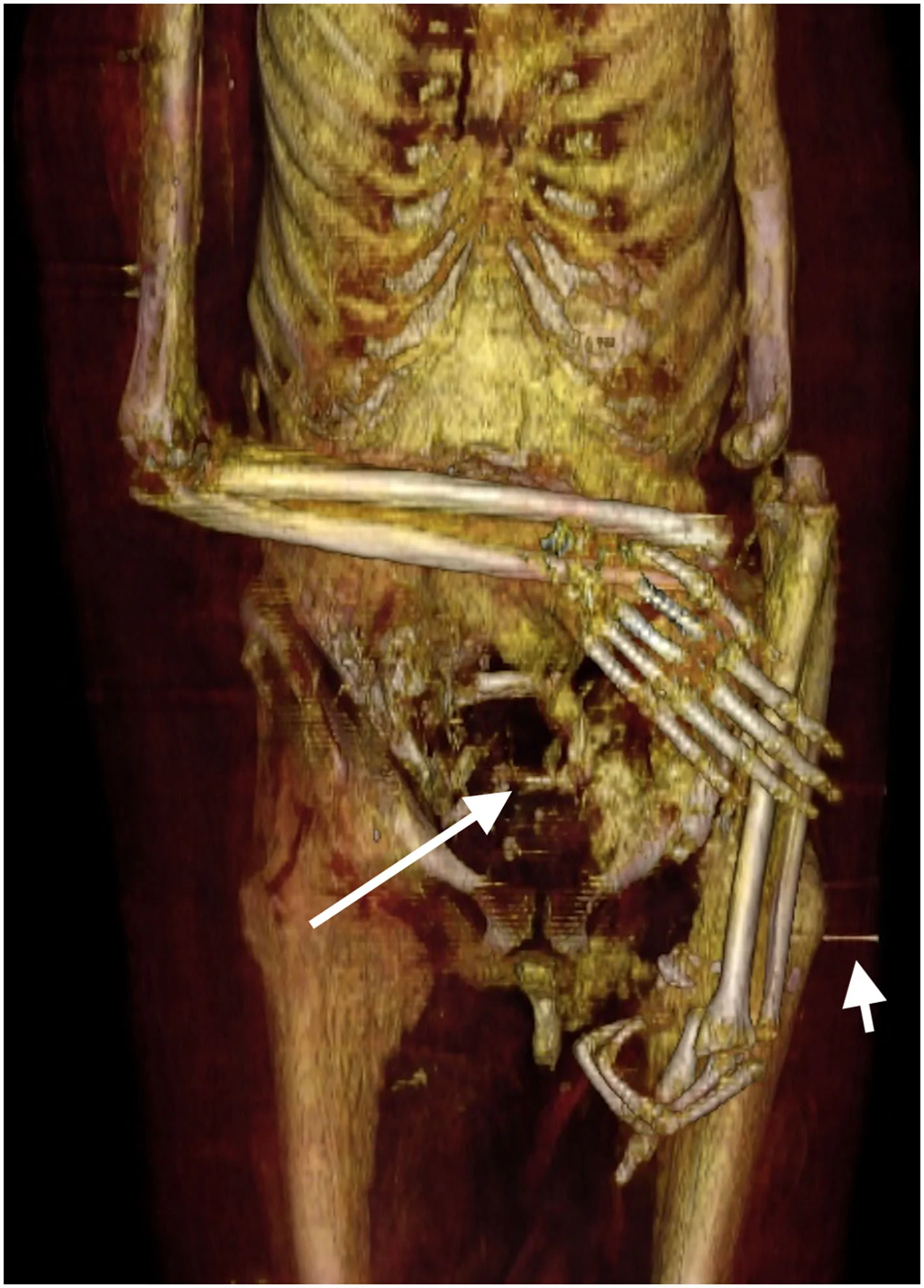
Three-dimensional frontal CT image of the lower torso and upper limbs of the mummy of Amenhotep I. The right forearm is flexed at the elbow and crosses the lower abdomen transversely; the right hand is dislocated at the wrist and is displaced anterior to the forearm. The dislocated left arm and forearm are placed extended along the left side of the body. The broken left (hand) has three flexed fingers; the missing two fingers are seen inside an anterior abdominal wall defect (long arrow). The fractures were likely inflicted by tomb robbers. The initial position of the arms was probably crossed in front of the body. A short pin is placed transversely across the bandages (short arrow) likely to fix the left disarticulated arm in place.

**Figure 6:**
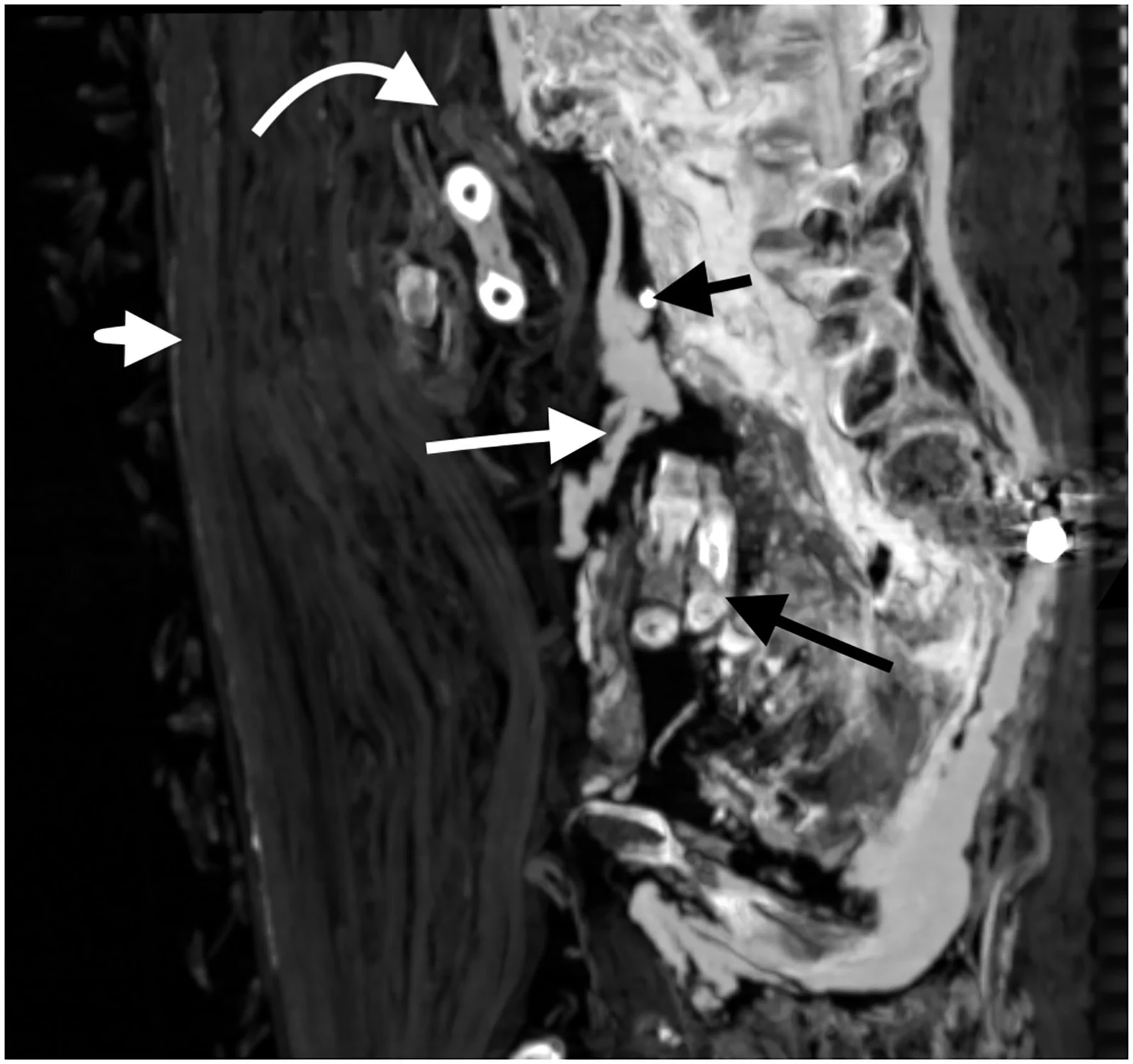
Sagittal CT image of the abdomen and pelvis of the mummy of Amenhotep I showing a large anterior wall defect. A linen band treated with resin (long white arrow) partly covers the defective anterior abdominal wall. An amulet is seen beneath the linen band (short black arrow). Two fingers missing from the left hand are placed inside the body cavity beneath the linen cover (long black arrow). The right forearm is individually wrapped (white curved arrow). The thickness of the wrapping is more in the front (short white arrow) than in the back of the body.

A large defect of the anterior wall of the abdomen and pelvis measures 120 × 180 mm in transverse and craniocaudal dimensions, respectively. The missing two fingers from the left hand are seen inside the abdominal defect (Figures 5, 6). The fractured medial lower part of the left pubic bone; the fracture edge is sharp with no evidence of bone healing.

**Figure 7:**
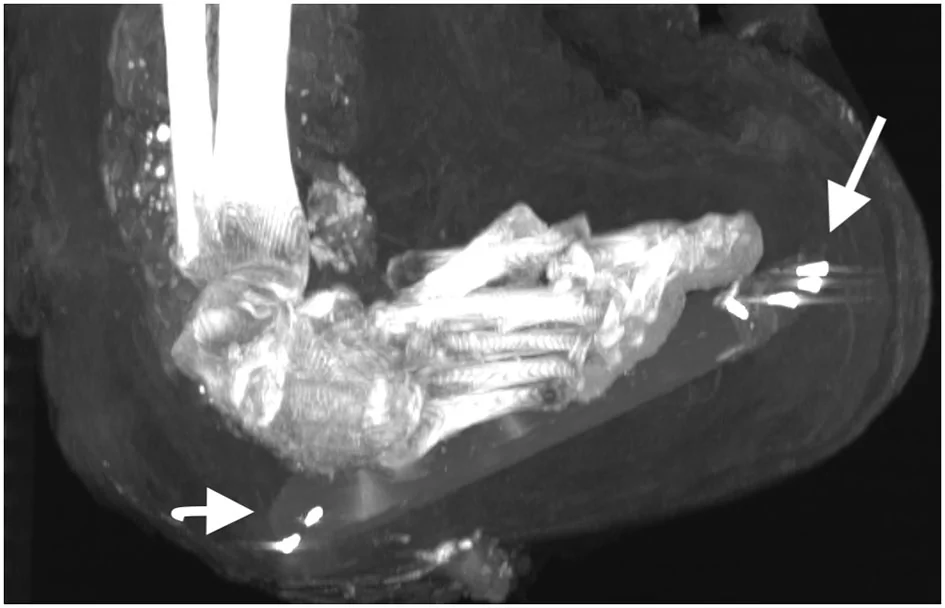
Sagittal thick slab three-dimensional CT image of the right foot of the mummy of Amenhotep I. The disarticulated bones of the right foot are placed on a wood board. Note that the metal nails in front (short arrow) and at the back (long arrow) of the board were likely placed to fix the position of the wood plaque to the surrounding wrappings.

Disarticulated bones of the right foot (Figure 7).

#### Age at Death

**Figure 8:**
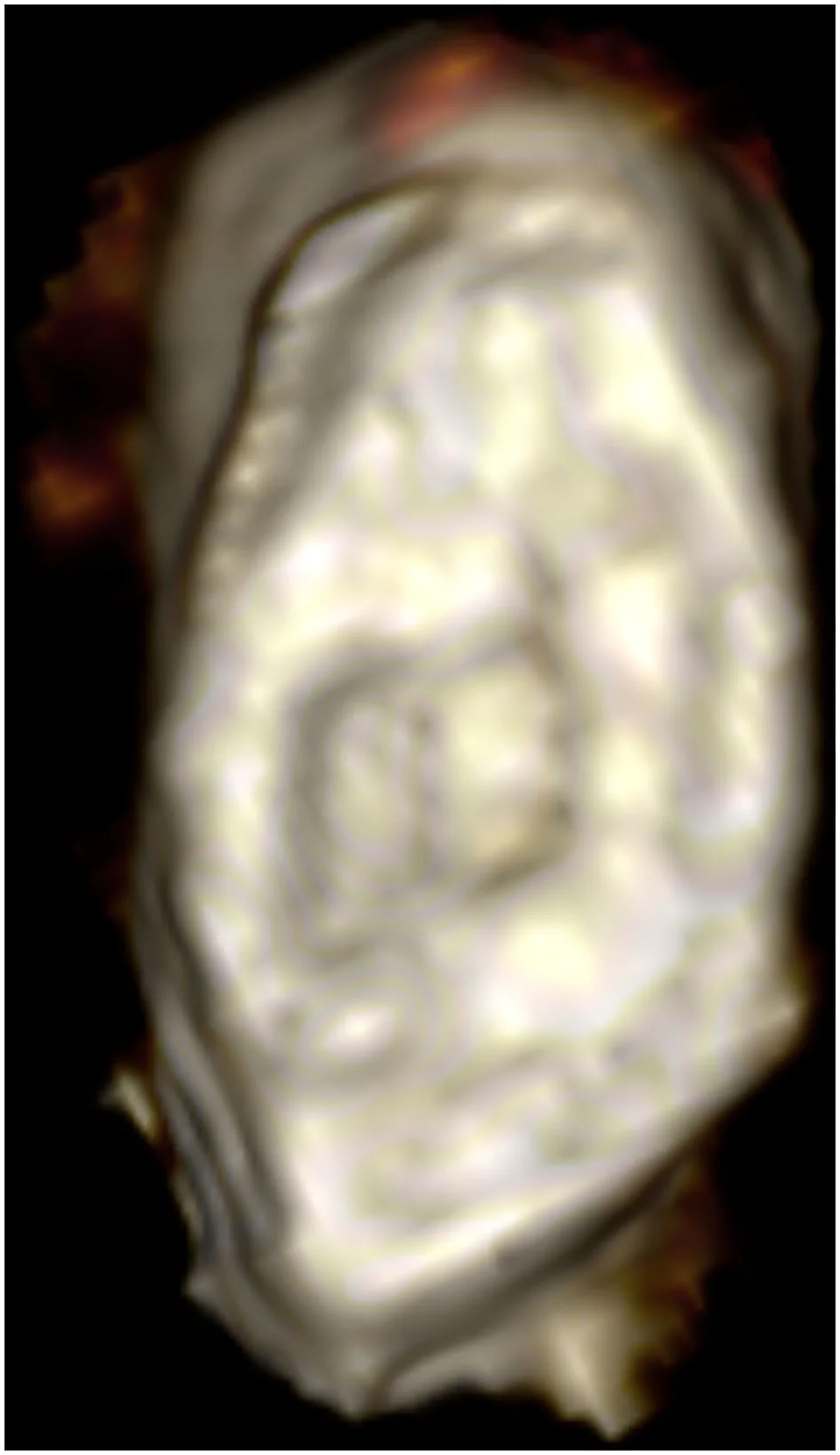
Three-dimensional CT image of the surface of the symphysis pubis of Amenhotep I the morphology indicates stage 4 corresponding to 35.2 ± 9.4 years old.

**Figure 9:**
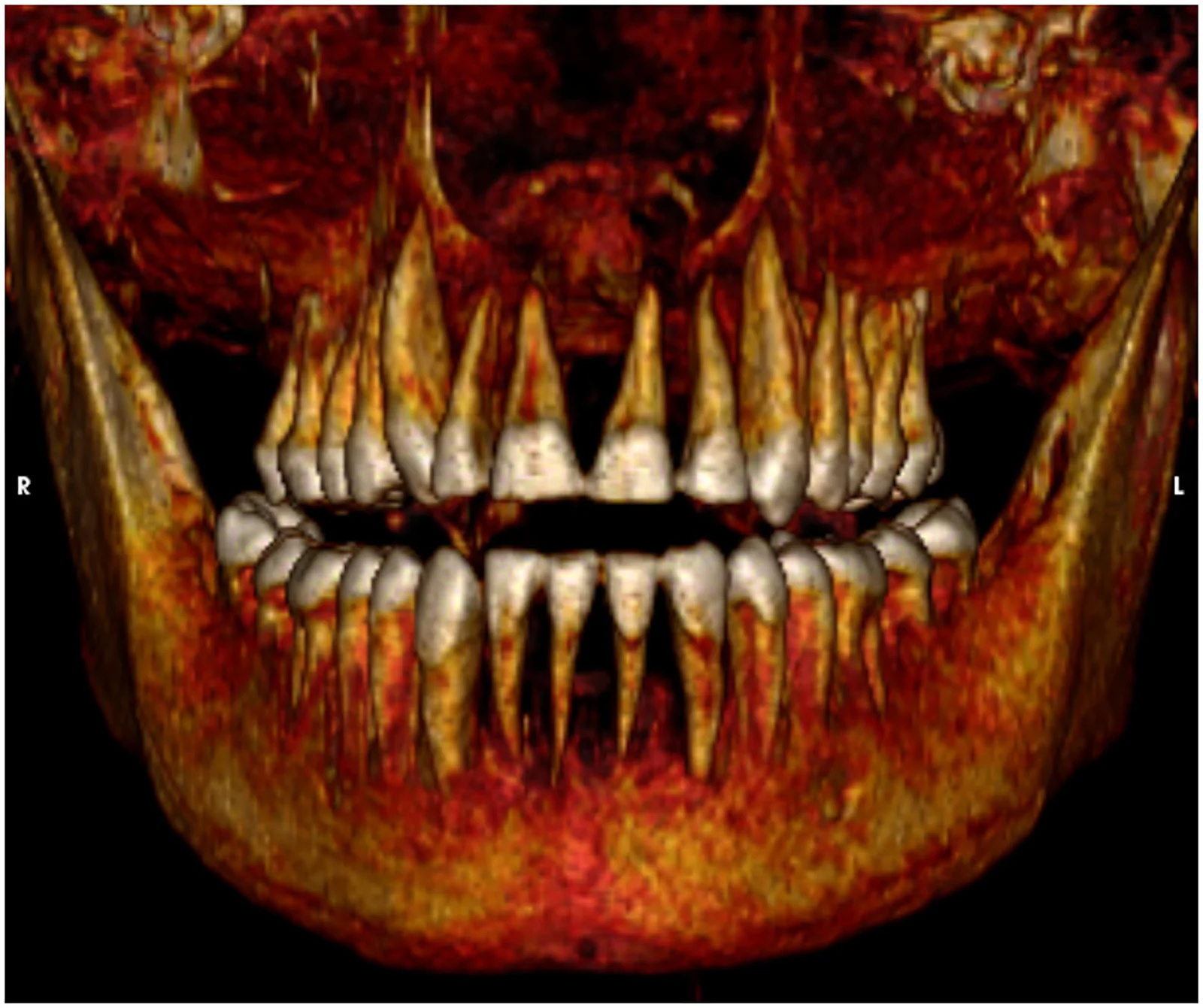
Three-dimensional CT image of the teeth of Amenhotep I in frontal view shows a full set of healthy teeth.

The age at death of Amenhotep I is estimated at 35 years based on the closure of epiphyses of all the long bones, as well as on the morphology of the surface of the symphysis pubis (stage 4 corresponding to 35.2 ± 9.4 years (Figure 8) (13). The mouth contains a complete set of teeth including all of the third molars (Figure 9). Mild attrition of the maxillary and mandibular teeth (13–15).

#### Stature

The vertex to heel length of the skeleton of Amenhotep I measures 161.5 cm in the sagittal reconstructed CT image. We measured the maximum length of the tibia (389 mm) and calculated the stature using the Raxter et al. regression equation for ancient male Egyptians: (Stature = 2.554 × 38.9 + 69.12 = 168.47 cm ± 3.002) (16).

#### Pathological Changes and Cause of Death

All the sets of teeth are available; there is minimal attrition without evidence of caries or remarkable periodontal disease. No CT evidence of bone diseases or joint degeneration. The penis shows evidence of circumcision. No cause of death could be detected in the CT images of the body.

#### Mummification

– Excerebration: The skull base is intact without evidence of an attempt to remove the brain (Figure 3). The shrunken brain occupies the posterior aspect of the skull cavity without evidence of any embalming materials (Figure 4).

– Evisceration: There is no evidence of viscera inside the body cavity. A vertical left flank opening measuring (90 mm × 58 mm × 51 mm in length, transverse, and craniocaudal dimensions, respectively) was likely the incision used for evisceration. The chest and abdominopelvic cavities are stuffed with loose linen (about −100 HU) and packs with variable CT densities consistent with linen treated with resin (70–120 HU). The heart is seen in the left hemithorax.

– Packing: There is no evidence of orbital packing. Traces of resin are noted on the desiccated eye globes. A small stopper of linen treated with resin is inserted in each nostril, which measures 16 mm × 5 mm in the right nostril, and 7 mm × 4 mm in the left. The desiccated tongue is seen at the back of the mouth; no resin or packs are seen within the mouth (Figure 4). No subcutaneous packing is noted in the face or elsewhere in the body.

– Wrapping: There is CT evidence of the individual wrapping of the right upper limb and the left lower limb; the fingers and toes are wrapped with the rest of the right hand and left foot, and not being individually bandaged. The disarticulated left upper limb has not been individually wrapped but bandaged along with the body and with the right upper limb. The right thigh and leg are individually wrapped. The disarticulated right foot has not been individually wrapped; it was bandaged to an underlying wood board and with the left foot. The circumcised penis has been independently wrapped. The body of the mummy is covered by transverse wrapping in a spiral fashion. The thickness of the mummy wrapping is (78–112 mm) in the front and (21–40 mm) at the back of the body.

– Arms position: The right forearm of Amenhotep I crosses the body with a right angle at the elbow. The disarticulated right hand is placed in front of the transversely oriented right forearm. The left upper limb is placed along the body side. The two missing fingers from the left hand are seen inside the abdominal cavity.

#### Amulets and Jewelry

A total number of 30 amulets/jewelry pieces are found in the wrapped mummy of Amenhotep I. The details of the amulets and jewelry are listed in Table 1.

**Table 1:** Computed tomography (CT) findings of Amulets and jewelry related to the mummy of Amenhotep I.

**Region**	**Number of amulets**	**Description and identification**
Right upper limb	7	– At the shoulder region (*n* = 3) Anterior to gleno-humeral joint: quartz/faience Eye of Horus (19 × 18 ×6 mm; 1,650 HU) Posterior to gleno-humeral joint: quartz/faience Eye of Horus (13 × 11 × 2 mm, 1,670 HU) Anterior to scapula: Quartz/faience eye of Horus (19 × 13 × 4.3 mm; 1,550 HU) – Between the wrapping at the back of the right arm (*n* = 3): At the upper arm: a gold Eye of Horus: (21 × 15 × 3 mm; 3,059 HU) At the lower arm: a gold Eye of Horus: (20 × 12 mm × 3; 3,070 HU) At the lower arm: quartz/faience scarab (11 × 9 mmx 3; 1,500 HU) – Between the wrapping at the back of the forearm (*n* = 1): An oblong shaped gold bead (7 × 3 × 4 mm; 3,069 HU)
Inside torso cavity	3	Behind the left sterno-clavicular joint (n=1): a rectangular quartz/faience amulet (7.5 × 3.5 mm; 1,790 HU) – Anterior abdominal wall behind embalming pack at L1 level in midline and to the left side (*n* = 2): an oblong quartz/faience bead (9 × 4 × 3 mm; 1,800 HU); a fired clay/faience scarab amulet (8.3 × 7.5 × 4 mm; HU 1,100 HU)
On torso surface and between wrappings	10	– In front of the right sterno-clavicular joint (*n* = 1): a discoid gold amulet (7 × 5 × 3mm; 3,000 HU). – Right posterior chest wall on outer wrapping (*n* = 2): a pointed gold amulet (7 × 4 × 3 mm; 3,066 HU); a rectangular gold amulet (6.5 × 4.5 × 3 mm; 3,069 HU). – At right lateral torso wall between wrappings from up to down (*n* = 5): a gold oblong bead (5 × 85 mm; 3,000 HU); a double plume quartz/faience amulet (19 × 12 × 5 mm; 1,789 HU); an oblong-shaped (Wadji) gold amulet (10 × 4.5 × 4 mm; 3,006 HU); a gold discoid amulet (5 × 4 × 2 mm; 2,979 HU); a pyramid quartz/faience amulet (10 × 10.4 × 9 mm); 1,800 HU). – At the left lateral side of pelvis on the body surface (*n* = 1): an oblong shaped amulet with pointed ends (snail shell) made of quartz/faience (45 × 20 × 17mm; HU 1,800 HU). – At the back of the pelvic region on the body surface (*n* = 1): A girdle formed of 34 beads gold beads (7.2 to 10.1 mm in diameter; 3,036 HU). The beads are joined with double metal strings at the periphery
Left lower limb	10	In front of the wrapping on the upper end of left femur arranged transversely from medial to lateral (*n* = 5): A rod-shaped stone amulet (18 × 7 × 6 mm; 1,993 HU); a rectangular plate quartz/faience (6.5 × 6.3 × 4mm; 1,360 HU); a rectangular plate fired clay amulet (8.6 × 8.3 × 6.4 mm; 1,100 HU); an oblong (Wadji) quartz/faience (10 × 5 × 3 mm; 1,370 HU); and a serpent head fired clay amulet (13 × 9 × 5 mm; 1,060 HU) On the outer wrapping at the medial of upper thigh (*n* = 1): an oblong fired clay amulet (12 × 8 × 5 mm; 1,300 HU) Within the wrappings at the back of the upper thigh (the general wrapping) (*n* = 1): a gold spherical bead (5 mm diameter; 3,028 HU) On the surface of the upper lateral leg (*n* = 1): a fired clay scarab amulet (7 × 7 × 5 mm; 1,400 HU) In the wrapping at the lateral of the lower leg (*n* = 1): a stone bead with a central hole (4.7 mm in diameter; 2,700 HU) In the wrapping at the medial of the lower leg (*n* = 1): an oblong-shaped quartz/faience amulet (6 × 3 × 3 mm; 1,900 HU).

#### Reburial Embalming Treatment

The mummy shows signs of repair:

– Fixing the detached head and fractured neck vertebrae: A linen band treated with resin wrapped the fractured cervical spine and kept the head and neck in line with the body (Figure 4).

– Covering a defect in the anterior abdominal wall:

A linen band treated with resin partly covers a defective anterior abdominal wall. Two amulets are seen placed beneath the linen band.

– Placement of the detached left upper limb alongside the body:

The left dislocated upper limb is placed alongside the left side of the body and has been wrapped with the body and the right arm.

– Resting the disarticulated right foot on a wooden board:

The disarticulated bones of the right foot have been placed on a wood board measuring 177 mm × 55mm × 7 mm in maximum length, breadth, and thickness respectively. The right foot and the wood board are wrapped together by linen layers. Six metal nails (9–10 mm long) are seen inserted in the wood board (four nailed in the front, and two in the back) likely to fix the position of the wood plaque to the surrounding wrappings.

– Using pins through the bandages along the disarticulated left arm:

Six pins (about 25–27 mm long and 2 mm in thickness) are placed transversely across the linen bandages on the left side of the body opposite the left lower forearm, likely to fix it in place. The CT densities of the pins range between −300 and 1,800 HU: four of these pins are at the lower end of the range (could be made of wood), and two pins at the higher end of the range could be made of ivory/bone.

**Figure 10:**
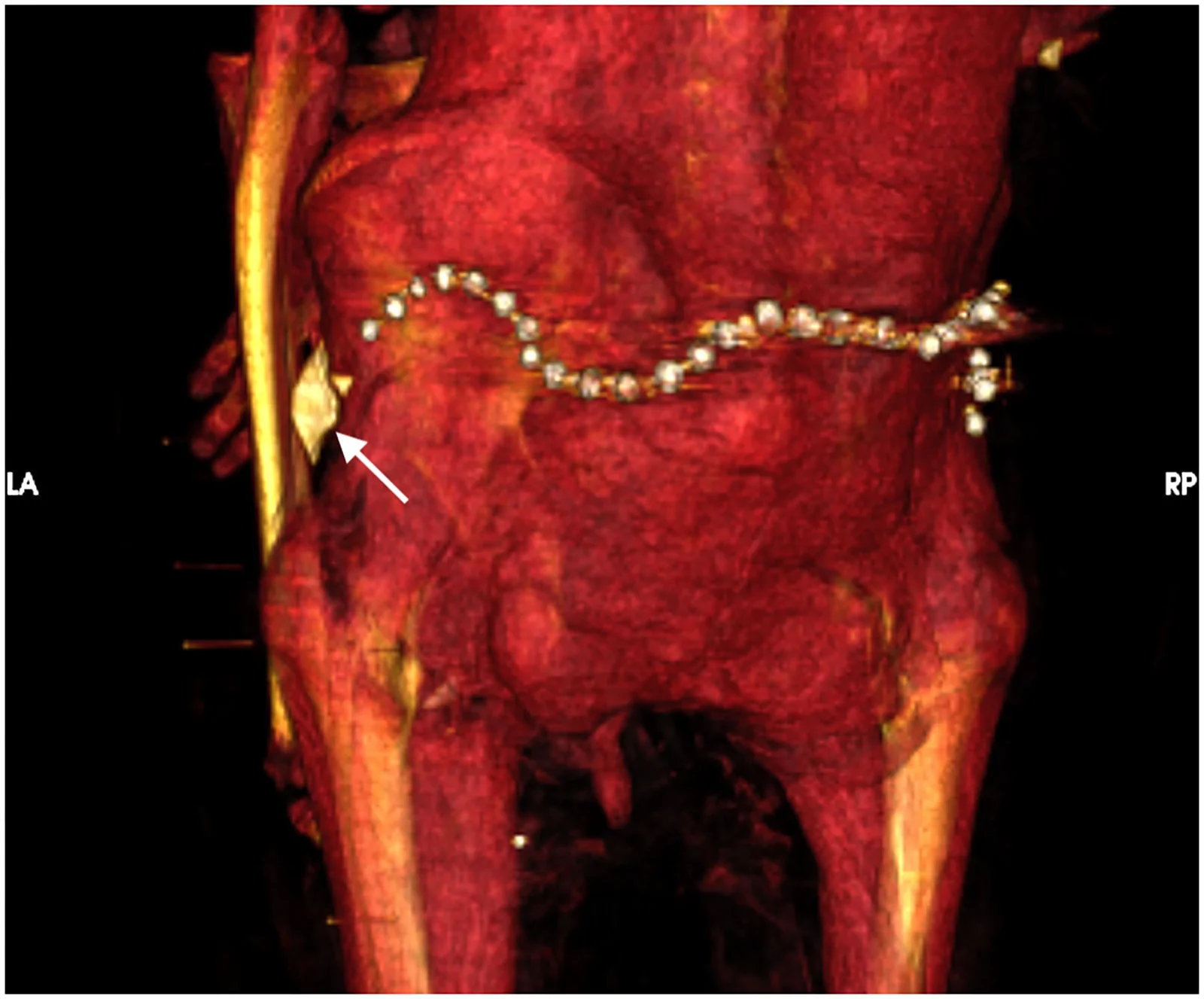
Three-dimensional CT image of the surface of the lower back of mummy Amenhotep I shows a beaded metallic girdle (likely gold) at the back of the pelvic region and a faience amulet in the shape of a snail shell (arrow) at the left hip region.

– Placement of amulets and jewelry:

The beaded girdle at the back of the pelvic region and the large amulet at the left hip region is placed on the surface of the mummy beneath the wrapping (Figure 10). Two amulets are placed beneath the linen band that covers the defective anterior abdominal wall.

#### Head Mask

A piece of cartonnage covers the head and neck of the wrapped mummy circumferentially and extends in a tripartite wig configuration till the mid-chest level in the front and back. The maximum dimensions of the cartonnage: in length is 444 mm in front and 399 at the back; in width is 320 mm; 225 mm in depth. The thickness of the cartonnage measures 5.5–6.5 mm; it shows a central low-density layer of linen/papyrus (−30 HU) covered by a thin layer of a higher density material (gesso plaster) from the outside and another layer of gesso from inside (700–1,000 HU).

**Figure 11:**
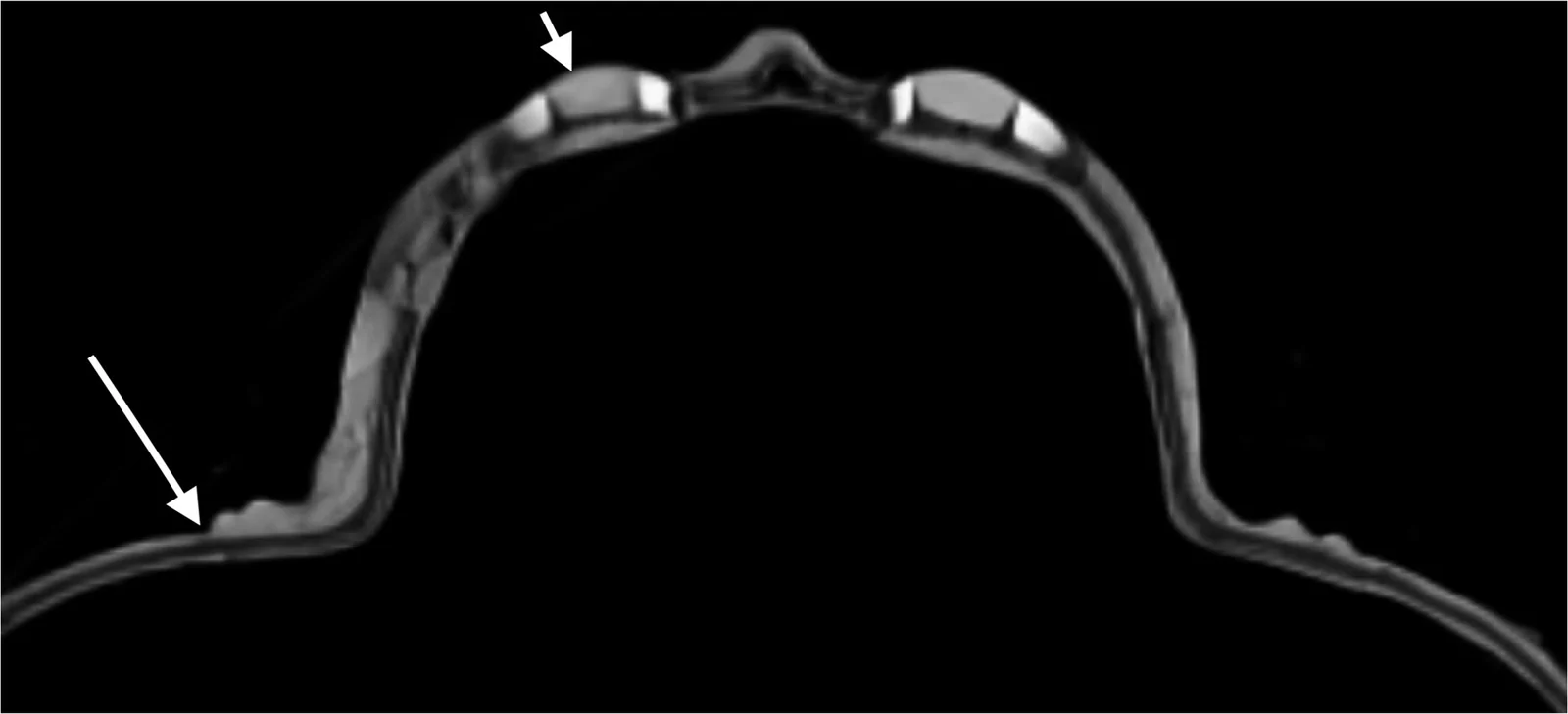
Axial CT image of the face mask at the level of the eyes. The face mask is mounted on the cartonnage (long arrow); the cartonnage is formed of a central low-density layer of linen/papyrus covered by denser layers of gesso (long arrow). Each eye of the mask is made of inlaid stones of a central discoid structure that corresponds to the black obsidian pupil and denser pieces on each side for the white of the eye likely made from quartz.

The face of the mask is formed of a separate piece of wood mounted on the molded front of the cartonnage. The wood piece measures 1,200 HU in CT density and measures: 250 mm in length, 184 mm in width, and 12 mm in thickness. The eyes of the mask are inlaid stones (Figure 11). The black pupil of each eye is made of a discoid structure with a homogeneous CT density (1,693–1,700 HU) corresponding to obsidian crystals. The black pupil measures (10.7 × 15.2 × 7.3 mm) and (10 × 16 × 7.2 mm) in width, length, and depth for the right and left eye pupils, respectively. The white of each eye is formed by two separate triangular pieces of a denser material (2,400 HU) placed on both sides of the black pupil measuring about 10–11 mm × 8.8–9.9 mm × 5.8–6 mm in length, width, and depth, respectively. On the forehead, there is a separately carved structure in the shape of a cobra; it measures 84 × 32 × 16 mm in length, width, and depth, respectively. The central material of the cobra measures −30 HU and is inlaid with stones of 2,200 HU. The cobra is inserted through a hole (7 mm in diameter) in the cartonnage with a stick (19 × 8 × 4 mm in length, width, and depth, respectively). The left side of the face mask shows a 120 mm long fissure fracture that starts just below the left eye and extends downward till the upper neck.

## Discussion

Unwrapping and dissection of mummies were often carried out in the past (3). These procedures are being condemned as they damage the ancient bodies and have been replaced by non-invasive imaging techniques such as CT (12). In this study, we used CT to digitally unwrap the mummy of King Amenhotep I.

The Thebes royal tombs were subjected to a series of tomb robberies (3). After being damaged by grave robbers, the mummy of Amenhotep I has been rewrapped twice in ancient times by the 21st Dynasty Priests (5, 6). The CT images show the extent of damage of the mummy of Amenhotep I that involved neck fractures and decapitation, a large defect in the anterior abdominal wall, and disarticulation of the extremities (left upper limb, right hand, and right foot). In addition to the injuries witnessed on the royal mummies, the plundering of the Theban necropolis was also documented in juridical documents known as “tomb robbery papyri”. The bulk of these documents were found in the temple of Medinet Habu and are concerned with the theft of the Thebes temples and tombs in the reign of Ramesses IX and Ramesses XI. The overall information gained from these archaeological documents is that the theft was often carried out by members of the burial group. The confession of thieves in the tomb robbery papyri indicates that they were after the jewels and amulets in between the wrappings and inside the bodies of the mummies (2). This explains that the postmortem injuries in the mummy of Amenhotep I targeted the neck and limbs, common places for jewelry, and hacked the abdomen wall in search of amulets inside the body cavity.

The height of the mummy of Amenhotep I measures 161.5 cm in the reconstructed CT images. However, the presence of multiple body fractures may affect the accuracy of the direct measurement of the height in CT images. We thus estimated the stature of Amenhotep I by measuring the length of a long bone (tibia). By using a regression equation for the stature of ancient male Egyptians (16), the height of Amenhotep I is 168.5 cm ± 3 cm. The CT study of the mummy of King Amenhotep I shows no pathological changes or a detectable cause of his death.

The plain X-rays done in 1967 by the Michigan University expedition estimated the age of Amenhotep I at death to be 20–25 years based mainly on the good teeth condition (11). This CT study enables a more accurate estimation of the age of death of the King based on a more reliable criterion: the 3D-morphology of the surface of the symphysis pubis (15). The pubic symphyseal surface becomes smoother with age, enabling the estimation of age (13). In this study, we estimated that the King probably died at the age of 35, about a decade older than X-ray estimation, which is more in accordance with the historical data that Amenhotep I reigned for 21 years (1, 2).

Mummification was practiced in ancient Egypt over a period of 30 centuries, aiming at preserving the dead body from decay for the resurrection according to their belief. The funerary rituals and mummification showed some variability in the different periods of the ancient Egyptian civilization (6). The royal mummies of ancient Egypt are an important resource for our understanding of the mummification techniques (3). CT imaging can help determine the mummification style through visualization of the bony and soft tissue elements as well as other foreign materials and packs (12).

The ancient Egyptians often removed the brain (excerebration) by introducing a tool into the nostril to break the weak part of the anterior skull base. The brain was removed and sometimes embalming materials were introduced inside the skull. Attempts of excerebration were as early as the Fourth Dynasty (18). The CT images of the mummy of Amenhotep I show that there was no attempt to remove the brain which is seen shrank and occupying the back of the skull. Other royal mummies dated around the time of Amenhotep I (late 17th Dynasty to early 18th Dynasty) have not been excerebrated (12). These include Seqenenre Taa II, Meritamun, Thutmose II, Thutmose III, and Hatshepsut (19–21). Excerebration became popular later in the 18th Dynasty and the peak of the procedure is suggested to be in the Ptolemaic Period (18).

To prevent body putrefaction, the ancient Egyptian embalmers removed the internal organs through an abdominal incision (6). The CT scan of the mummy of Amenhotep I revealed a vertical left flank incision. Before the 18th Dynasty, incisions were vertical in the left flank and extended from the anterior superior iliac spine upwards. An example of a vertical left flank incision has been reported in the CT of the mummy of Seqenenre Taa II (17th Dynasty) (12, 20). Later in the 18th Dynasty, most of the royal mummies have oblique incisions parallel to the left inguinal ligament as in Thutmose III (18th Dynasty), Ramesses II (19th Dynasty), and Ramesses III (20th Dynasty) (12). During the process of evisceration, the heart was preserved (6). The CT images identified the presence of the heart within the chest of Amenhotep I. Similarly, previous CT studies identified the preserved heart in other New Kingdom royal mummies as in Thutmose II, Ramesses II, Ramesses III (12). The ancient Egyptian embalmers used different materials to fill the emptied body cavity (6, 12). The CT images show that the body cavity of Amenhotep I, similar to other royal mummies of the New Kingdom, is filled with materials with different CT densities representing linen fibers, and linen packs treated with resin (12). To make the corpse look lifelike, the embalmers of the royal mummies dated to the New Kingdom usually used packs in the eyes, nose, mouth, and under the skin (22). However, this study shows that the mummy of Amenhotep I has not received such treatment.

Bandaging and wrapping the mummified body with linen sheets was an important stage of the embalming process in ancient Egypt (2). The bandaging techniques of the mummy changed during the different periods of ancient Egyptian Civilization. In the Middle Kingdom, a large sheet of linen was used to wrap the mummy. In the New Kingdom, the wrapping became more elaborate as the embalmers used overlapping spiral bands to wrap each limb individually. Once all the body parts were wrapped, the embalmers started wrapping the body as a whole. As each part of the mummy was bandaged, the embalmers uttered spells and placed protective amulets on the body and the different linen layers. In later periods, wrapping grew more sophisticated as the bodies were wrapped with narrow bandages, forming complex patterns (8). The bandages carried the name or titles of the deceased (2). Little information is known about the style of wrapping of the New Kingdom royal mummies since most of them were found in cached burials in Deir el Bahari and Amenhotep II tomb in the Valley of the Kings after being rewrapped in the 21st Dynasty. The only non-cached burial royal mummy known is Tutankhamun; unfortunately, the wrappings had become carbonized, and little is known about its pattern (8). The CT images show that the mummy of Amenhotep I has been fully wrapped. We suggest that some bandages could be original, dated to the 18th Dynasty in addition to the reburial work in the 21st Dynasty. The CT images show that the right upper limb and the left lower limb are individually wrapped and have amulets on the surface of the limb or in between the layers of the bandages. While the detached left upper limb and the disarticulated right foot have no individual bandages or related amulets and have been wrapped with the body and/or the contralateral limb. We assume that the individual wrapping of these limbs was original, dated to the 18th Dynasty, and was a little disturbed by the tomb robbers. While the disarticulated left upper limb and right foot have lost their original individual bandages and amulets and were not individually wrapped but only offered general bandaging with the rest of the body by the 21st Dynasty priests. The circumcised penis has individual wrapping.

The right forearm of Amenhotep I crosses the body with a right angle at the elbow. The preservation of the individual wrapping and amulets of the right upper limb may support the hypothesis that the forearm was crossed at the time of the initial mummification in the 18th Dynasty. The disarticulated left arm and forearm were likely placed alongside the body during the reburial in the 21st Dynasty. The crossing forearms on the body are commonly seen in the New Kingdom royal mummies. The mummy of King Ahmose, who preceded Amenhotep I, has both arms along the body (6). This makes the mummy of Amenhotep I likely to be the first to appear with crossed forearms in the New Kingdom followed by other Kings such as Thutmose II, Thutmose III, and Tutankhamun (12).

The embalmers used funerary objects to protect the deceased. Specific amulets and their placement were described in the ancient Egyptian documents, The Book of the Dead (8). The CT examination of the mummy enables the non-invasive study and determination of the presence of amulets and jewelry (12). The multiplanar and 3D reconstruction of the CT images of the wrapped mummy of Amenhotep I show the precise location, measurement, and identification of 30 amulets and jewelry: seven amulets in the bandages of the right upper limb, 10 amulets within the bandages of the left lower limb, and 13 amulets/jewelry related to the torso. Ten out of the 13 amulets/jewelry related to the torso were on the body surface or between the wrappings; only three amulets were inside the body cavity: heart amulet inside the chest cavity and two amulets in the abdomen. An amulet can be many things; the power of an amulet was in its shape, material, and color. The type of material used to make the amulet depended on the wealth of the deceased (8). The CT densities of the amulets/jewelry enabled the identification of its material composition (17). The CT densities of the amulets of Amenhotep I suggest that they are made from different materials: 11 metal amulets and jewelry (likely golden), 13 quartz/faience amulets, five fired clay amulets, and one stone amulet. Funerary amulets have different shapes. The CT images of the mummy of Amenhotep identified a variety of amulets including Wadjet (Eye of Horus), scarab, double-plume, Wadji (papyrus scroll), beads, snail-shell, serpent-head, ib (heart scarab), and other amulets of non-specified identity. The maximum length of the amulets found in mummy Amenhotep I varied between 45 and 5 mm.

Ancient Egyptian mummies from the Predynastic Period onward were provided with jewelry constructed of various materials ranging from shells and beads to gold and gemstones (8). Some of the best-known and most elaborate pieces of jewelry were found on the intact royal mummy of Tutankhamun (3). However, fewer jewelry pieces were found in the royal mummies of the New Kingdom found in cached burials. Previous CT studies of the royal mummies of the New Kingdom found in the two royal caches identified Thutmose III (BC) to be wearing two golden bracelets hidden behind the wrappings (17). In this study, the CT images show a belt formed of 34 gold beads placed directly on the lower back of the mummy of Amenhotep I.

The wrapped mummy of Amenhotep I provides a unique opportunity to understand the intervention done during its reburial by the 21st Dynasty priests. The hieratic dockets written in ink on the coffin gave information on the historical process of the reburial of the mummy Amenhotep I (2). The CT examination suggests how the Theban priests of the 21st Dynasty repaired the injuries of the mummy of Amenhotep I inflicted by the tomb robbers and restored the royal dead. The reburial treatment included: fixing the detached head and fractured neck vertebrae with a resin-treated linen pad; covering the defective anterior abdominal wall with a linen band treated with resin; placement of the detached left upper limb extended alongside the body and placing the two detached fingers of the left hand inside the abdomen; resting the disarticulated right foot on a wooden board with metallic nails; wrapping the body; using pins (likely made of ivory or bones) to fix the bandages at the disarticulated left upper limb and right foot. Metal nails and wooden pins were used in the manufacture of funerary furniture and coffins in ancient Egypt (23). Although nails have been known since the Old Kingdom, they were not used in woodwork until the 18th Dynasty. During the Old Kingdom, the only metal available was copper. From the Middle Kingdom (c. 2160 BC to 1788 BC), bronze and copper were present. However, iron has not been used in funerary equipment until 700 BC (24).

During the reburial of the mummy of Amenhotep I, we suggest that the 21st Dynasty embalmers placed two amulets beneath the linen band they used to cover the anterior abdominal wall defect. We suggest that during reburial the embalmers placed or maintained in place a golden beaded girdle at the back of the pelvis.

The mummy of Amenhotep I wears a mask. Mummy masks were part of an elaborate burial ritual in ancient Egypt. They are usually made of cartonnage. Cartonnage is a cardboard-like material that was used to make coffins for mummies. The cartonnage substance is made of glued linen or papyrus, coated with plaster and water, then molded to the desired shape. The dry cartonnage could be then colored or gilded. Cartonnage could be made as one piece that covers the full mummy, or in smaller pieces to cover certain regions such as the head, pectoral, or legs. Cartonnage was used in the different periods of Egyptian ancient civilization dating back to the end of the Old Kingdom (2686–2181 BC). Cartonnage became popular in the Third Intermediate Period (1069–664 BC), as well as later in the Ptolemaic-Early Roman Periods (330 BC−250 AD) (6). A CT scan can study a mummy cartonnage (25). In this study, the CT images show the shape and full dimensions of the mask of Amenhotep I which is partly hidden by the overlying garlands. The layers of the cartonnage have different CT densities based on their material; the linen/papyrus layer has a lower CT density than that of the plaster (gesso) layers on both the inner and outer surfaces. The CT images can help to understand how the parts of the head mask were assembled. The CT scan shows the face of the mask as a separate piece of wood mounted on the front of the cartonnage. The face of the mask has been individualized to the facial features identical to that on the coffin of Amenhotep I (1). The facial features of the mask of Amenhotep I have given a life-like appearance by using eyes inlaid with stones. The black eye pupil is discoid in shape made of Obsidian, a naturally occurring glass; while the eye white is formed with two separate pieces made from a denser material (likely quartz) and are placed on both sides of each pupil. Different manufacturing methods and materials have been identified in the inlaid eyes in ancient Egyptian collections including obsidian, rock crystal, quartz, and ivory (6, 26). The manufacturer of the inlaid eye in the mask of Amenhotep I used three separate pieces of stones which likely required more work than other methods where the white eye was made of one piece with a tapering hole in the middle to receive a disc representing the pupil (26). The CT images identify the good status of preservation of the cartonnage with no cracks or bent areas. However, the wood facial mask shows a long thin fissure fracture; a piece of information that may help in its restoration.

The embalmers covered the mummy with garlands of red, yellow, and blue flowers recognized as Delphinium orientale, Sesbania egyptiaca, Acacia nilotica, and Carmanthus tinctorius (8). It was the beautiful reburial job offered by the 21st Dynasty priests for the mummy of Amenhotep I that made Maspero decide not to unwrap the mummy or disturb its novelty (8). The reburial project of the New Kingdom Royal mummies by the 21st Dynasty priests was accused to be for the intention of the removal and reuse of the royal burial equipment for the Third Intermediate Period Kings. Examples include the reemployment of two coffins originally made for Thutmose I (18th Dynasty) for King Pinudjem I (21st Dynasty) (2). However, this CT examination of the mummy of Amenhotep I reveals how the Theban priests of the 21st Dynasty had lovingly restored the royal mummified body of Amenhotep I and preserved or provided rich amulets and jewelry. This study may make us have re-confidence in the goodwill of the reburial project of the Royal mummies by the 21st Dynasty priests.

In this study we used CT to scan the mummy of King Amenhotep I. CT is considered the diagnostic gold standard imaging modality for studying mummies. Magnetic resonance imaging has a limited value in the examination of dry mummified remains as this modality primarily provides information on the location of mobile hydrogen within the body. Future analysis of the mummy of Amenhotep I may include dual-energy CT scanning. Different materials have different linear attenuation coefficients at different energy levels. Dual-energy CT scanning uses two different energy levels that may help to identify and characterize the embalming materials in the mummy. Dual-energy scanning can also help to reduce metal artifacts in CT images induced by golden amulets and jewelry placed on the mummy (27).

The specialized CT imaging technique in this study may have applications in paleo-anthropological and bio-archaeological studies of mummies from Egypt as well as other cultures such as Peru (28). CT is being used nowadays more frequently in forensic medicine. Postmortem CT imaging can provide valuable information in identifications in mass disasters, trauma, and homicidal cases (27). The methodological tool in this study may have potential applications in studying cadavers that have been preserved by natural mummification due to extreme environmental conditions such as dry hot deserts or freezing mountains (27, 28).

## Conclusion

The digital unwrapping of the mummy of Amenhotep I using CT sets a unique opportunity to reveal non-invasively the physical features of the King, understand the mummification style early in the 18th Dynasty, and recognize the reburial intervention done in the 21st Dynasty.

## Data Availability Statement

The datasets presented in this article are not readily available because the data collected during the current study are available from the authors on reasonable request and with permission of the Egyptian Ministry of Antiquities and Tourism. Requests to access the datasets should be directed to saharsaleem1@ymail.com.

## Author Contributions

SS was responsible for the conception and design, acquisition of data, analysis and interpretation of data, as well as drafting of the manuscript, and generation of the figures and accountable for the accuracy and integrity of the work. ZH made substantial contributions to the design, interpretation of the results, and revision of the intellectual content and agreed to be accountable for the integrity of any part of the work. All authors read and approved the manuscript.

## Conflict of Interest

The authors declare that the research was conducted in the absence of any commercial or financial relationships that could be construed as a potential conflict of interest.

## Publisher's Note

All claims expressed in this article are solely those of the authors and do not necessarily represent those of their affiliated organizations, or those of the publisher, the editors and the reviewers. Any product that may be evaluated in this article, or claim that may be made by its manufacturer, is not guaranteed or endorsed by the publisher.

## Correction note

This article has been corrected with minor changes. These changes do not impact the scientific content of the article.

## Abbreviations

BC: Before Christ
cm: centimeter
C: cervical vertebra
CT: Computed Tomography
D: Dorsal vertebra
FOV: Field-Of-View
HU: Hounsfield Unit
kVp: Kilovolt peak
mAS: milliampere-seconds.
